# Structural basis of mutant-selectivity and drug-resistance related to CO-1686

**DOI:** 10.18632/oncotarget.18588

**Published:** 2017-06-21

**Authors:** Xiao-E Yan, Su-Jie Zhu, Ling Liang, Peng Zhao, Hwan Geun Choi, Cai-Hong Yun

**Affiliations:** ^1^ Institute of Systems Biomedicine, School of Basic Medical Sciences, Peking University Health Science Center, Beijing 100191, China; ^2^ Department of Biophysics, School of Basic Medical Sciences, Peking University Health Science Center, Beijing 100191, China; ^3^ Department of Biological Chemistry & Molecular Pharmacology, Harvard Medical School, Boston, MA 02115, USA; ^4^ Department of Cancer Biology, Dana-Farber Cancer Institute, Boston, MA 02115, USA

**Keywords:** NSCLC, EGFR kinase, T790M, CO-1686, structural pharmacology

## Abstract

Non-small-cell lung cancers (NSCLCs) caused by activating mutations in the kinase domain of epidermal growth factor receptor (EGFR) initially respond to first-generation reversible drugs gefitinib and erlotinib. However, clinical efficacy is limited due to the development of drug-resistance that in more than half of the cases are driven by the secondary T790M mutation. CO-1686 is one of the third generation irreversible inhibitors that inhibits EGFR activating mutants, including those with concurrent T790M, while avoiding the off-target toxicity owing to inhibition of wild-type EGFR in treating EGFR mutation-positive NSCLCs. Despite the remarkable success, the experimentally determined structure of this agent in complex with EGFR T790M remains unknown. In this study, we determined crystal structures of EGFR T790M or L858R mutants covalently bound by CO-1686. Based on these structural data, we can explain why CO-1686 irreversibly inhibits EGFR and selectively prefers T790M, which may help improving this or similar compounds, and explain why EGFR L718Q and L844V mutations incur resistance to this agent.

## INTRODUCTION

Lung cancer is the leading cause of cancer related deaths, which accounts for nearly one third of all cancer deaths worldwide [1]. Despite prolonged research and clinical prevention strategies, the 5-year survival rate of lung cancer is less than 20% in patients in the United States (http://seer.cancer.gov). The two major types of lung cancers are non-small-cell lung cancer (NSCLC) and small-cell lung cancer, which account for about 85% and 15% of all lung cancers, respectively [2].

Activating mutations in the kinase domain of epidermal growth factor receptor (EGFR) are one of the major causes of NSCLCs, among which the most frequently seen mutations are the single site mutation leading to a leucine-to-arginine substitution at residue 858 (L858R) in exon 21, and the deletion mutation in exon 19 resulting in loss of the pentapeptide ELREA (delE746-A750). Patients harboring these typical activating mutations respond very well to therapy with the 4-anilinoquinazoline based reversible EGFR tyrosine kinase inhibitors (TKIs), such as gefitinib and erlotinib (the first-generation drugs) [3–6].

Unfortunately, despite dramatic initial response to the targeted therapy, EGFR mutation-positive patients usually suffer from the development of drug resistance and tumor progression after 9 to 14 months of treatment. In approximately 50-60% of the relapsed cases the drug-resistance are driven by a secondary point mutation in EGFR that leads to a threonine to methionine substitution at residue 790, the gatekeeper residue (T790M) [7–9]. Previous structural and mechanistic studies showed that EGFR T790M enhances the affinity of the kinase for ATP, leading to the reduction of the efficacy of any ATP-competitive inhibitors. In consequence new agents to counter EGFR T790M drug-resistance mutation must overcome the reinforced ATP binding affinity conferred by T790M [10]. The second-generation irreversible EGFR TKIs such as neratinib (HKI-272) had been expected to inhibit T790M-mutant EGFR with the capacity of competing off ATP by covalent binding to EGFR. However, these TKIs were effective only in *in vitro* or preclinical studies but not in clinical trials, probably due to the potent inhibition of wild-type EGFR accounting for adverse toxic effects [11, 12].

**Figure 1:**
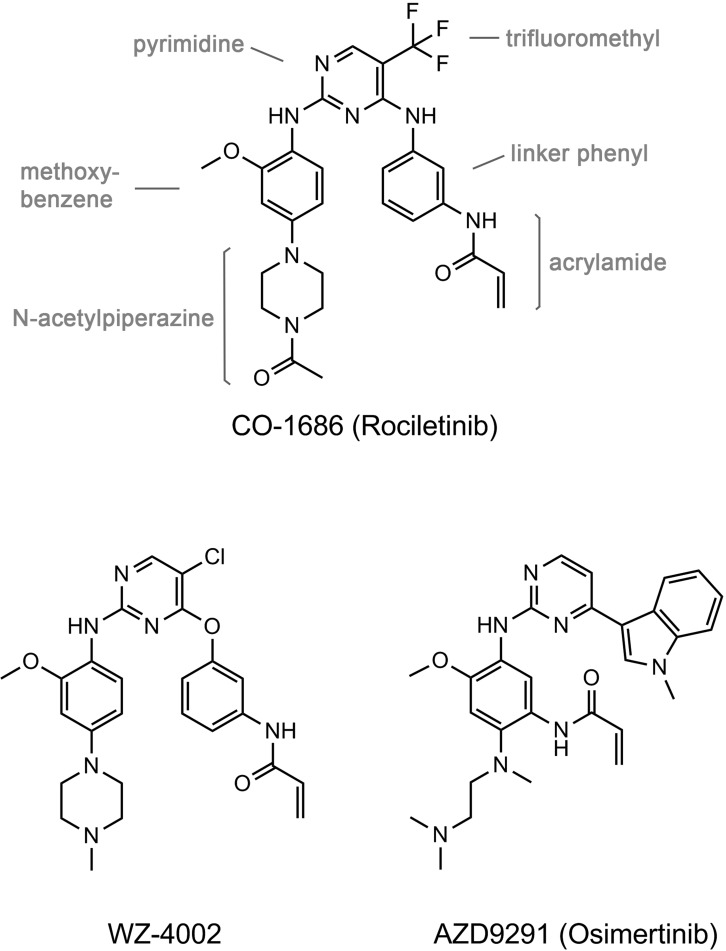
Chemical structures of the third generation EGFR TKIs discussed in this report

To resolve this issue, novel EGFR TKIs have been developed to selectively inhibit EGFR activating mutations with concomitant T790M while sparing the wild-type EGFR. The third generation pyrimidine-based irreversible EGFR TKIs (Figure 1), such as WZ4002, AZD9291 (osimertinib) and CO-1686 (rociletinib), not only effectively inhibit EGFR T790M, but also are much less potent in inhibiting wild-type EGFR and other kinases, are therefore expected to reduce side effects compared to afatinib [13–15]. The recent studies showed that both AZD9291 and CO-1686 exhibited excellent clinical efficacy in NSCLC patients harboring EGFR T790M, with more than 50% response rates, meanwhile skin and gastrointestinal toxicities are also less than those typically observed for the first generation EGFR TKIs [16–18]. In order to elucidate the binding mode of the compound to EGFR, to understand the structural basis of its specificity toward the T790M mutation, and to learn the drug-resistance mechanism conferred by several newly identified CO-1686-resistant mutations, we conducted the structural pharmacological studies on EGFR T790M and L858R mutants with CO-1686.

## RESULTS

### Overall crystal structures of EGFR T790M or L858R in complex with CO-1686

**Figure 2:**
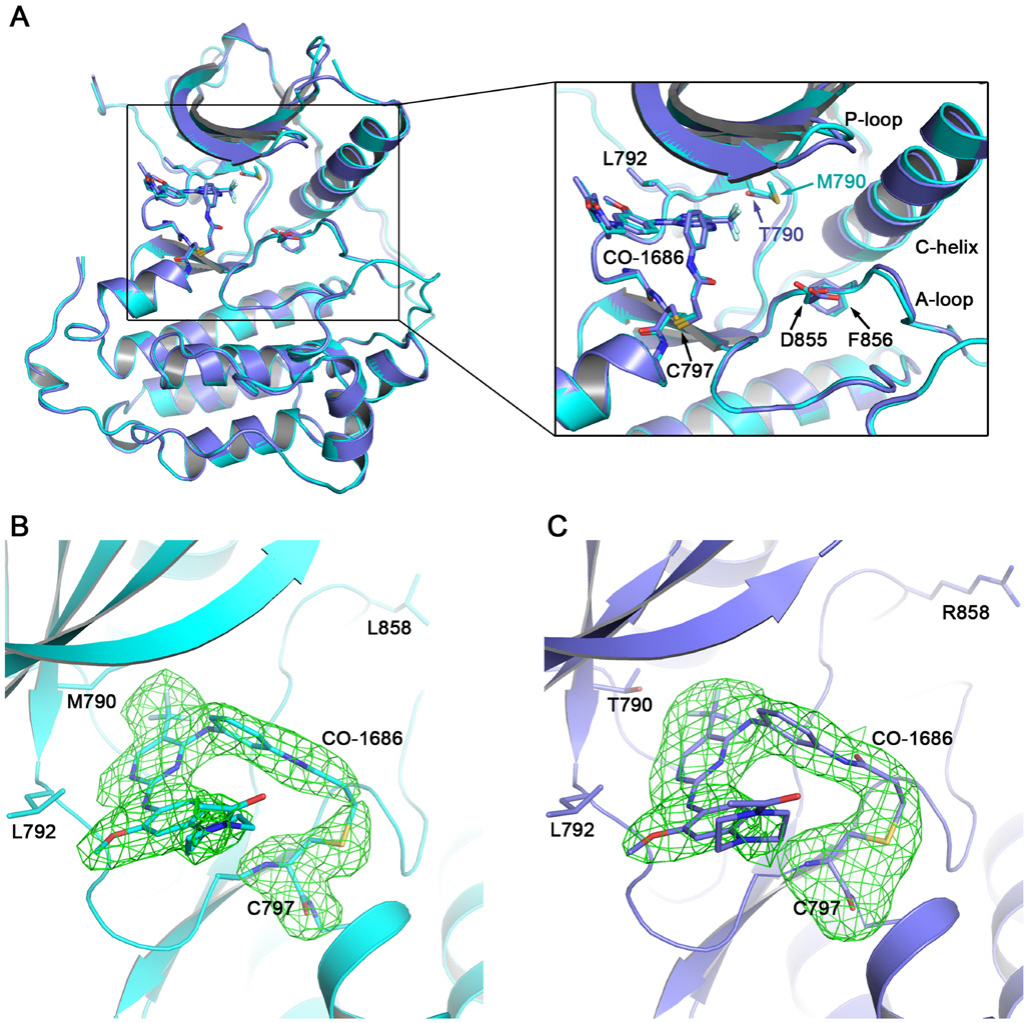
Overall EGFR/CO-1686 complex structure and covalent linkage between the compound and the kinase **(A)** Superimposition of T790M/CO-1686 and L858R/CO-1686 complex crystal structures. **(B)** The Fo-Fc omit map of CO-1686/Cys797 in the T790M/CO-1686 complex crystal structure. **(C)** The Fo-Fc omit map of CO-1686/Cys797 in the L858R/CO-1686 complex crystal structure. The EGFR T790M and L858R mutant proteins are shown as cyan and slate cartoons, respectively. CO-1686 and the key amino acid residues discussed in this report are shown as sticks with their carbon atoms colored in the same way as the protein. The Fo-Fc omit maps are contoured at 2.5σ and shown as green meshes.

CO-1686 is currently in phase I/II clinical trials in NSCLCs harboring EGFR activating mutations [14]. Despite the exciting safety and efficacy of CO-1686 in human clinical trials, the experimentally determined structure of this agent in complex with EGFR kinase remains unclear. Walter, A.O. *et al*. proposed a theoretical model of CO-1686 in complex with EGFR T790M based on the previously reported crystal structure of EGFR T790M/WZ4002 complex [13, 14]. However, it still remains doubtful if this theoretical model really illustrated the true structure of the T790M/CO-1686 complex. We therefore determined the complex crystal structures of EGFR T790M/CO-1686 and EGFR L858R/CO-1686 (Figure 2).

**Figure 3:**
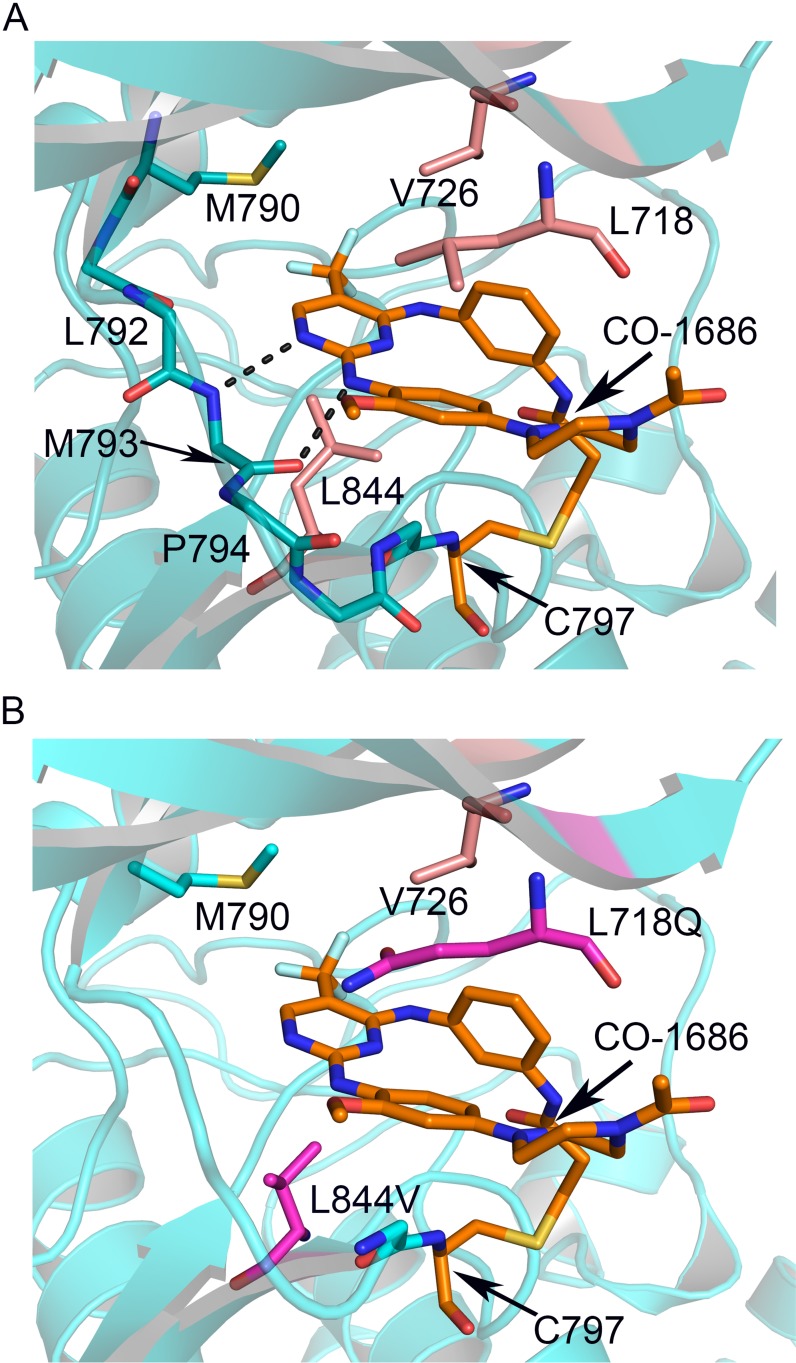
Interactions between CO-1686 and EGFR and the structural basis of drug-resistance conferred by L718Q and L844V **(A)** Crystal structure of CO-1686 in complex with EGFR T790M. The EGFR kinase is shown as cartoons in cyan, and the bound CO-1686 is shown as sticks in orange. The amide and carbonyl atoms of Met793 interact with the aminopyrimidine of CO-1686 through hydrogen bonds shown by dashed lines. Residues contacting CO-1686 are shown as sticks. **(B)** Structural modeling illustrating the influences of EGFR L718Q and L844V mutations to the interactions with CO-1686. The L718Q mutation (carmine) is predicted to hinder the binding of CO-1686 owing to steric hindrance and/or abolishment of hydrophobic interaction, while the shorter side chain of L844V (carmine) will weaken the hydrophobic interaction with the pyrimidine core of CO-1686.

The overall structures of these two complexes are highly similar to each other. One EGFR protein molecule is observed in the asymmetric unit of both crystal structures. The binding modes of CO-1686 to EGFR T790M and L858R are essentially the same and EGFR mutant proteins adopt the “DFG-in/C-helix in” active conformation (Figure 2A). As expected, CO-1686 covalently binds to EGFR through Cys797 in both structures (Figure 2B, 2C). The solution exposed N-acetylpiperazine moiety of the compound is coplanar to the methoxybenzene moiety and it bends towards Leu718 and wraps its side-chain in the structures of T790M/CO-1686 and L858R/CO-1686, which would facilitate the binding of the compound to EGFR (Figure 3A). It was predicted to be perpendicular to the methoxybenzene moiety in the theoretical model [14], which may not be reasonable since this conformation would incur too close contact between the piperazine and the Leu718 side-chain.

### Structural basis for CO-1686 specificity

In the T790M/CO-1686 and L858R/CO-1686 complex crystal structures, the anilinopyrimidine core of CO-1686 form two hydrogen bonds with Met793 amide and carbonyl in the hinge of the kinase (Figure 3A). The methoxyl substituent extends towards Leu792 side-chain. This methoxyl substituent in CO-1686 likely plays a key role in the superior selectivity of CO-1686 towards EGFR since other kinases with a cysteine residue equivalent to Cys797 in EGFR (such as Jak3 and the TEC-family kinases) usually harbor a bulkier residue (tyrosine and phenylalanine, respectively) at the position equivalent to Leu792 that would hinder the binding of the compound due to steric hindrance with the methoxyl.

In the structure of T790M/CO-1686, the trifluoromethyl (−CF_3_) substituent attached to the pyrimidine ring contacts the mutant gatekeeper residue Met790 by hydrophobic interaction, which may be the only difference between the T790M/CO-1686 and L858R/CO-1686 structures. The hydrophobicity afforded by the T790M gatekeeper mutation is beneficial to the potency of this drug towards EGFR. A wild-type gatekeeper residue (Thr790) would not afford this beneficial interaction with the compound (such as that observed in the L858R/CO-1686 structure), which explains why this agent prefers binding to the T790M mutant.

### Structural basis for drug-resistance conferred by L718Q and L844V

Despite the excellent efficacy of CO-1686 in clinic treatment, it is inevitable that a number of patients will eventually develop acquired drug resistance after long-term drug administration. Recently, the data showed that Cys797 was the most common site of secondary mutations (C797S and C797G) mediating resistance to WZ4002, CO-1686, and AZD9291; and secondary mutations L718Q and L844V appeared frequently in WZ4002 and CO-1686 resistant models [19].

In the determined EGFR T790M/CO-1686 and L858R/CO-1686 complex crystal structures, multiple hydrophobic interactions were found to facilitate the binding of the compound to EGFR. The pyrimidine core of the compound forms a hydrophobic interaction with Leu844 primarily; the methoxybenzene moiety forms a hydrophobic interaction with Leu718; while the linker phenyl ring carrying the acrylamide warhead of the compound into proximity to the thiol of Cys797 forms hydrophobic interactions with both Leu718 and Val726 side-chains (Figure 3A). Therefore the hydrophobic residues Leu718, Val726 and Leu844 all play essential roles in the binding of the compound to EGFR. This observation can explain the recent finding that L718Q and L844V cause resistance to CO-1686 [19]. The L718Q mutation would destroy the beneficial hydrophobic interaction with the methoxybenzene of the compound due to steric hindrance and/or loss of hydrophobicity. The L844V mutation though retains hydrophobicity, the side-chain of Val is too short to make significant hydrophobic interaction with the pyrimidine core of the compound (Figure 3B).

### Comparison between EGFR T790M/CO-1686 and T790M/WZ4002 complex crystal structures

**Figure 4:**
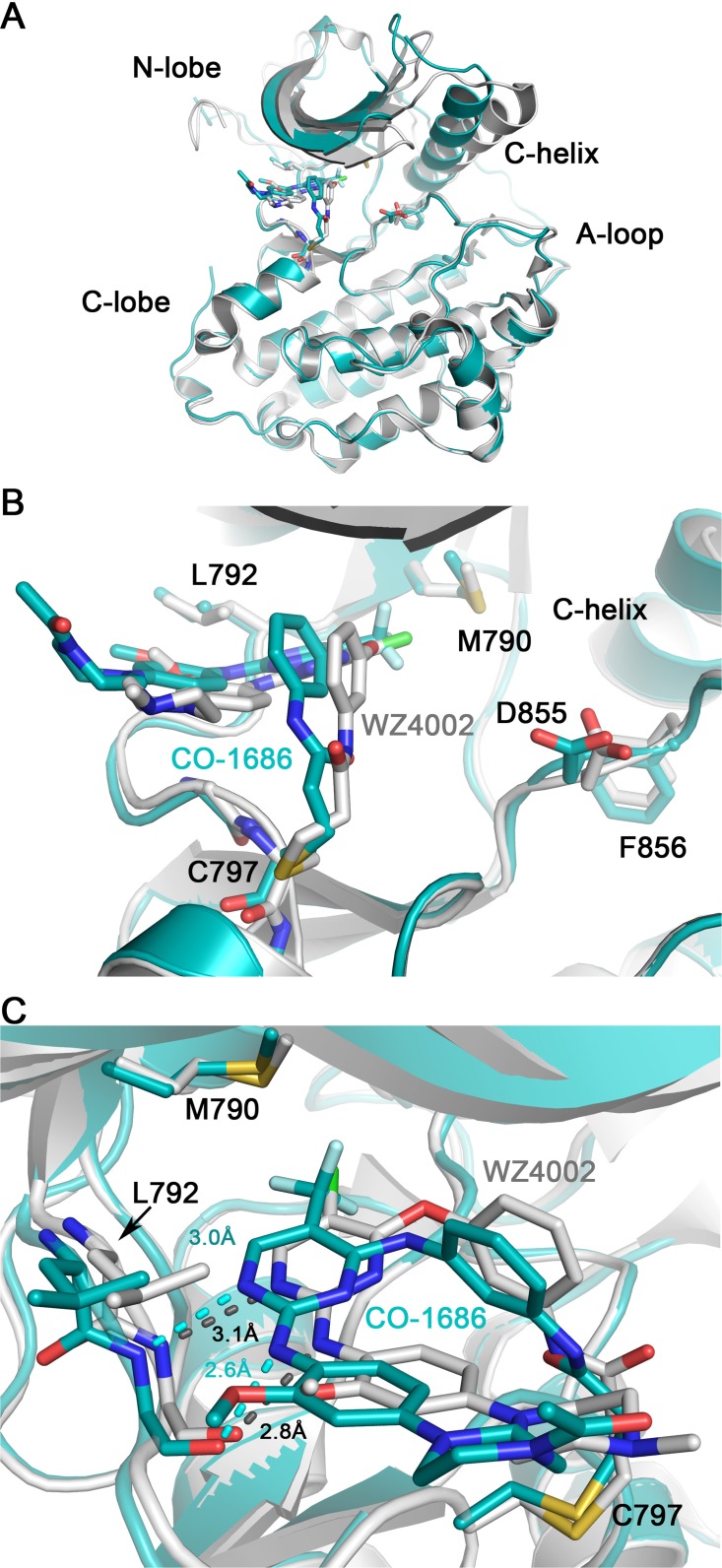
Comparison of T790M/CO-1686 and T790M/WZ4002 complex crystal structures Superimposition of the T790M/CO-1686 and T790M/WZ4002 overall structures is shown in panel **(A)**, while the side-view and top-view of the ATP binding pocket are show in panel **(B)** and **(C)**, respectively. The EGFR kinase in the CO-1686 and WZ4002 complex crystal structures are shown as cyan and gray cartoons, respectively. The compound and key residues are shown as sticks colored in the same way as the protein. The hydrogen bonds between the anilinopyrimidine core and Met793 main-chain amide and carbonyl are indicated by dashed lines. The lengths of these hydrogen bonds are labeled.

WZ4002 is another third-generation mutant-selective covalent inhibitors targeting EGFR T790M [13]. Although the binding modes of CO-1686 and WZ4002 to EGFR are almost the same (Figure 4A), there remain subtle differences between them. Probably because the trifluoromethyl substituent on the pyrimidine ring contacting the mutant gatekeeper residue Met790 is much bulkier than the chlorine substituent in WZ4002, the CO-1686 core scaffold is “pushed out” a bit compared to WZ4002 (Figure 4B), and the distance of the bidentate hydrogen bonds between the anilinopyrimidine core and the ‘hinge’ residue Met793 are closer in the T790M/CO-1686 structure than those observed in the T790M/WZ4002 structure (Figure 4C). What’s more, the C-helix in T790M/CO-1686 structure rotates about 20° inwards when compared with the T790M/WZ4002 complex (Figure 4A). However, since the two complex crystal structures, T790M/CO-1686 and T790M/WZ4002, were made in different ways (co-crystallization versus soaking) and the crystallization conditions were different, these differences might simply be caused by the methodology and/or crystallization condition differences.

## DISCUSSION

In this study we determined the crystal structures of EGFR T790M or L858R in complex with the third-generation drug CO-1686, which provide insights into why CO-1686 displays preference towards the EGFR T790M drug-resistant mutation while sparing the wild-type EGFR. In the T790M/CO-1686 complex structure, the anilinopyrimidine scaffold of CO-1686 fits well to the methionine gatekeeper. The hydrophobic interaction between the trifluoromethyl moiety and the Met790 side-chain enhances the affinity of CO-1686 towards T790M. The methoxyl group is necessary for the favorable selectivity of the compound to EGFR against other kinases bearing bulkier residues in the position equivalent to Leu792 in EGFR. Previously data showed that selectivity of CO-1686 to EGFR T790M/L858R versus wild-type EGFR is >12-fold and versus other kinases is 20- to 2000-fold [20]. *In vivo*, studies with human tumor xenografts also confirmed remarkable antitumor activity as well as selectivity over wild-type EGFR [14, 20]. Significantly, irreversible kinase inhibitors, such as WZ4002, rely on covalent bond formation for potent inhibition [13].

Our crystal structures help illustrate the reasons why L718Q and L844V are resistant to CO-1686. Since the chemical structure of CO-1686 is highly similar to that of WZ4002, and their binding modes to EGFR are highly similar to each other, the same drug-resistance mechanism should apply to WZ4002, too. Since Leu718 and Leu844 both directly contribute to CO-1686/WZ4002 binding through hydrophobic interactions, any mutations to eliminate these hydrophobic interactions would interfere with the drug binding. It is well known that mutations are a common mechanism of drug resistance to kinase inhibitors. The L844V mutation has been previously detected in a NSCLC patient [21]. Moreover, EGFR L718P, L718V, and L718M mutations have been described in clinic [22–24]. According to our model, all these mutations may weaken the binding affinity of CO-1686/WZ4002. However, since CO-1686 and WZ4002 are ATP-competitive inhibitors, whether these mutations incur resistance to these agents needs to be further investigated. For example, if these mutations also weaken ATP binding, they may not result in drug-resistance.

In summary, our crystallographic data provided insights into the structural basis of the selectivity of CO-1686 towards EGFR T790M, which may be helpful for future improvement of this or similar compounds. We observed the hydrophobic interactions between Leu718/Val726/Leu844 and CO-1686, which can explain why L718Q and L844V are resistant to CO-1686/WZ4002, and help predicting other potential drug-resistance mutations.

## MATERIALS AND METHODS

### Cloning and expression of EGFR 696-1022 mutants

Construct spanning residues 696-1022 of the human EGFR and harboring the L858R mutation was generated as previously described [25]. Construct of the human EGFR kinase domain (residues 696-1022) harboring the T790M mutation was generated from the cDNA of wild-type EGFR by site-directed mutagenesis and cloned into the pFastBac HTA vector (Invitrogen). A 6x-His tag followed by a TEV protease cleavage site was fused to the N-terminus of the EGFR protein to facilitate later purification of the protein. Transfection, virus generation and amplification were carried out in sf9 insect cells according to the official protocol of the Bac-to-Bac Baculovirus expression system (Invitrogen). The EGFR mutant kinases were then expressed in sf9 insect cells.

### Purification of EGFR 696-1022 mutants

Cell pellets were suspended in lysis buffer (20 mM Tris-HCl, 150 mM NaCl, 5 mM KCl, 20 mM imidazole, 1 mM tris(2-carboxyethyl) phosphine hydrochloride, pH 8.0) supplemented with protease inhibitor mixture (Complete EDTA-free, Roche) and lysed by sonication. The lysate was centrifuged at 20,000 rpm for one hour at 4°C, then the supernatant was incubated with Ni-NTA Sepharose beads (GE Healthcare). The beads were washed with wash buffer (20 mM Tris-HCl, 500 mM NaCl, 1% Glycerol, 1 mM TCEP, 20 mM imidazole, pH 8.0) and then the protein was eluted with the same buffer supplemented with 300 mM imidazole. The eluted protein was concentrated to 1 mL and incubated with His-tagged TEV for 4 hours at 4°C. After that, the uncleaved fusion protein and the His-tagged TEV were removed by diluting the sample with wash buffer to 20 mM imidazole and passing it through the Ni-NTA Sepharose beads (GE) for the second time. The flow-through containing only the untagged EGFR proteins were then concentrated to 0.5 mL. The mutant proteins were further purified by size-exclusion chromatography (Superdex 200) in the wash buffer and was concentrated to 15 mg/mL. Aliquots were made and flash frozen in liquid nitrogen and stored in −80°C refrigerator.

### Crystallization

The apo-L858R crystals were obtained by hanging drop vapor diffusion using 0.5 μl of protein (4 mg/ml in 20 mM Tris, 500 mM NaCl, 1% glycerol, 1 mM TCEP) and 0.5 μl of reservoir solution containing 0.1 M HEPES pH 7.8, 40% PEG400, 0.15 M NaCl, 5 mM TCEP. Crystals of CO-1686/L858R were made by soaking the apo-L858R crystals for 4 hours at 20°C in the reservoir solution supplemented with 1 mM CO-1686. The EGFR T790M/CO-1686 complex crystals were prepared by co-crystallization. The EGFR T790M protein (15 mg/mL in 20 mM Tris, 500 mM NaCl, 1% glycerol, 5 mM TCEP) was pre-incubated with 1 mM CO-1686 on ice for 4 hours before setting up the crystallization tray. The initial crystals were obtained by sitting drop vapor diffusion against the reservoir solution of 0.0665 M HEPES pH 7.5, 1.1 M tri-sodium citrate at 20°C. The best crystals for data collection were grown by hanging drop vapor diffusion using 0.5 μl of protein and 0.5 μl of reservoir solution containing 0.07 M HEPES pH 7.5, 0.9 M tri-sodium citrate, 5 mM TCEP at 20°C. For data collection, all crystals were rapidly dipped in reservoir solution supplemented with 25% ethylene glycol and flash frozen in liquid nitrogen.

### Structure determination

X-ray diffraction data were collected on beamline ID-19 at Advanced Photo Source (APS) at 100K at Argonne National Laboratory or beamline BL19U1 at Shanghai Synchrotron Radiation Facility (SSRF). The diffraction data were processed using HKL3000 [26]. The structures were determined by molecular replacement with Phaser [27] utilizing the publicly available EGFR L858R/ANP structure (PDB ID 2ITV) as the searching model for the L858R/CO-1686 and EGFR T790M structure (PDB ID 2JIT) as the searching model for T790M/CO-1686. Repeated rounds of manual refitting and crystallographic refinement were then performed using COOT [28] and Phenix [29]. Topology and parameter files for the inhibitor were generated using PRODRG [30]. Diffraction data and refinement statistics are summarized in Table 1. The atomic coordinates and structure factors for T790M/CO-1686 and L858R/CO-1686 have been deposited in the Protein Data Bank with entry IDs 5XDK and 5XDL, respectively.

**Table 1:** Data collection and refinement statistics of the EGFR/CO-1686 complex crystal structures

	CO-1686/EGFR T790M	CO-1686/EGFR L858R
**Data collection**		
Space group	*I*23	*I*23
Cell dimensions		
*a*, *b*, *c* (Å)	145.5, 145.5, 145.5	143.5, 143.5, 143.5
α, β, γ (°)	90.0, 90.0, 90.0	90.0, 90.0, 90.0
Resolution (Å)	50-2.35(2.43-2.35)	50-2.70(2.91-2.70)
* R*_p.i.m_.^a^ (%)	3.3(54)	3.5(3.49)
*I*/σ*I*	27.6(2)	25.98(2.19)
Completeness (%)	100(100)	98.3(99.5)
Redundancy	16.7(16.3)	7.3(7.4)
** Refinement**		
Resolution (Å)	41.99-2.35	38.36-2.7
No. reflections	20894	13462
* R*_work_/*R*_free_^b^	0.217/0.250	0.208/0.252
No. atoms		
Protein	2456	2348
Water	108	19
*B*-factors		
Protein	40.86	75.72
Water	42.12	73.59
R.M.S. deviations		
Bond lengths (Å)	0.016	0.011
Bond angles (°)	1.307	1.122
Ramachandran plot		
Favored, %	98.67	97.89
Allowed, %	1.33	2.11
Disallowed, %	0	0

*Values in parentheses are for highest-resolution shell. One crystal was used for each data set.

^a^$R_{p.i.m.}=\sum_{h}(\frac{1}{n_{h}-1})\sum_{l}|I_{hl}-\langle I_{h}\rangle |\sum_{h}\sum_{i}\langle I_{h}\rangle$, where *I_hl_* is the *l*th observation of reflection *h* and 〈*I_h_*〉 is the average intensity for all observations *l* of reflection *h*. *R*_p.i.m_. is a multiplicity-independent R factor to evaluate diffraction data quality [31].

^b^ The R factor for refinement is defined as:
$$R=\sum ||F_{obs}|-|F_{cal}||/\sum \left|F_{obs}\right|$$
where *F_obs_* and *F_cal_* are observed and calculated structure factor amplitudes, respectively. *R*_work_ is calculated using reflections included in the refinement, while *R*_free_ is calculated using reflections excluded from the refinement.
